# Post-Intensive Care Syndrome—10 Years after Its Proposal and Future Directions

**DOI:** 10.3390/jcm11154381

**Published:** 2022-07-28

**Authors:** Shigeaki Inoue, Nobuto Nakanishi, Kensuke Nakamura

**Affiliations:** 1 Department of Disaster and Emergency and Critical Care Medicine, Kobe University Graduate School of Medicine, Kusunoki-cho 7-5-2, Chuo-ward, Kobe 650-0017, Japan; nobuto_nakanishi@yahoo.co.jp; 2 Department of Emergency Medicine, Teikyo University School of Medicine, 2-11-1 Kaga, Itabashi-ku, Tokyo 173-8606, Japan; mamashockpapashock@yahoo.co.jp

With the development of intensive care medicine and the standardization of various therapeutic guidelines and education systems, mortality rates in critically ill patients have improved markedly. In sepsis, mortality decreased by 52.8% from 1990 to 2017 [1]. In acute respiratory distress syndrome, the mortality rate decreased from 35.4% to 28.3% from 1996 to 2013 [2]. However, patients leaving the intensive care unit (ICU) continue to have physical, cognitive, and psychiatric problems for several years after discharge. Many patients never regain their pre-hospital activities of daily living (ADL) and still have a difficult path back to society. Post-intensive care syndrome (PICS) is a general term for long-term physical, cognitive, and mental disorders that occur during or after ICU stay, affecting not only the long-term prognosis of ICU patients but also the mental health of their families [3,4]. The current Special Issue, “Post-intensive care syndrome,” in the Journal of Clinical Medicine, is dedicated to collecting high-quality scientific contributions that mainly focus on its epidemiology, prevention, and therapeutic strategies.

The epidemiology of PICS has been extensively documented over the past few years, and the full picture of PICS is becoming clearer. Almost half of the critically ill adult patients had worsened ADL status after ICU discharge. Aging, a high burden of chronic illness, and a longer duration of mechanical ventilation were risk factors for worsened ADL status [5]. In a prospective study, published last year in Japan (J-PICS study), the incidence of PICS in patients who were admitted to the ICU at 6 months was 64%, with 32% having physical impairment, 38% cognitive impairment, and 15% mental impairment. Surprisingly, 18% of patients had PICS in more than one area, indicating that PICS is a complex condition [6]. The results of the study also showed that PICS is a complex condition. In addition, 87% of patients admitted to the ICU with novel coronavirus disease (COVID-19) had physical disability, 8% had cognitive disability, 48% had mental disability at 1 month, and 50% had cognitive disability at one month [7]. At 4 months, 50% had cognitive impairment, 31% anxiety, 21% depression, 54% insomnia, and 14% post-traumatic stress disorder (PTSD) [8]. The review article in this Special Issue demonstrated the pathophysiology of COVID-19 and the recent data on PICS after COVID-19 infection [9].

One of the most prominent symptoms of PICS is physical impairment. Physical impairment is an important problem to be prevented and managed because it significantly reduces quality of life (QOL)—as do cognitive and psychiatric impairments [3]—and it is observed in one-third of patients 6 months after ICU discharge [6]. Long-term physical disability occurs in approximately 30% of ICU survivors after 3 to 6 months in critically ill patients [6,10]. In recent years, several biomarker studies on PICS physical disability and ICU-acquired weakness have been reported. Urinary titin, a giant sarcomere protein, which functions as a spring for muscle extension and elasticity, could be a biomarker of muscle atrophy and physical impairment of PICS [11,12,13].

Cognitive impairment occurs in approximately 40% of ICU survivors after 3–6 months of critical illness [6]. Cognitive impairment, including impaired long-term memory, attention, language, decision making, and executive functioning, is thought to be related to a variety of mechanisms, including metabolic abnormalities, cerebral ischemia, excessive inflammation, disruption of the blood–brain barrier, oxidative stress, and microglial activation [14].

Mental disorders include depression, anxiety, PTSD, and sleep disorders. About one-third of ICU patients experienced a psychiatric disorder one year after discharge from the ICU, including 28% with depression, 17% with anxiety, and 6% with PTSD [15]. These mental health problems negatively impact patients’ health-related quality of life [16]. A recent prospective observational study found that 35% of ICU patients still had one of these mental disorders 3 months after discharge [17].

In addition, one of the important risk factors for PICS is frailty [18]. Critically ill patients had frailty in 19% of patients prior to ICU admission. Compared with patients without frailty, patients with frailty had significantly increased hospital mortality and delirium rates and significantly prolonged ICU and in-hospital length of stay [19]. Furthermore, in oral heath, occlusal insufficiency had a significant impact on PICS. Malocclusion on ICU admission was associated with ADL loss in critically ill patients. Furthermore, poor oral health was a poor prognostic factor among critically ill patients [20]. Based on these findings, dental care and follow-up will be essential for the prevention of PICS in the future.

Critical illness significantly impacts the quality of life of the patient’s family, who experience anxiety, depression, and PTSD during the patient’s ICU stay and after discharge. This condition has been termed PICS-F (Family). PICS-F was present in 48% of families approximately 90 days after the ICU stay, with 13% having depression, 29% anxiety, and 39% PTSD [21]. Sepsis survivors have a high prevalence of PTSD. Sepsis survivors have high healthcare utilization and related costs that persist after discharge from the hospital [22]. Since this economic impact after severe illness is one of the causes of PICS mental disorders and PICS-F, the establishment of financial support should be needed in the future.

PICS evaluations vary widely. It is recommended by the Society of Critical Care Medicine (SCCM) that PICS be evaluated within 2 to 4 weeks of discharge from the hospital as the initial time of evaluation and continue to be evaluated in subsequent periods [23]. The article in this Special Issue describes the details of their evaluation method [9]. In several assessments of PICS, grip strength is one of the useful evaluation tools that also reflects mental status and QOL [24].

In the ABCDEF bundle as PICS prevention [9], early mobility has been the most studied. Early rehabilitation by physicians or therapists beginning within 3 days was safe with low mortality and improved physical function in patients who underwent coronary artery bypass grafting [25]. Furthermore, early mobilization was associated with decreased psychiatric symptoms 3 months after hospital discharge in critically ill patients [26]. In patients with coronavirus disease pneumonia, an early mobilization program prevents muscle weakness and decreases psychiatric disorders [26].

Thus, 10 years after the proposal of PICS [4], the epidemiology, treatment, and other aspects of PICS are becoming clearer. Fujinami et al. established a mouse model of PICS that is a hybrid of a mouse model of sepsis and animal behavior analysis [27]. The model is based on a molecular model of PICS, and it would be one of the strategies to elucidate the molecular mechanisms of PICS.

As mentioned above, PICS has a significant impact on the lives of patients and their families and imposes a tremendous burden on society. The year 2022 will mark exactly 10 years since the advocacy of PICS. We hope that this opportunity to review the latest findings on PICS will help as many ICU survivors as possible to overcome PICS and return to their normal lives.
